# 2015 Relaunch as Open Access *Pediatric Neurology Briefs*

**DOI:** 10.15844/pedneurbriefs-29-1-1

**Published:** 2015-01

**Authors:** John J. Millichap, J. Gordon Millichap

**Affiliations:** 1 Division of Neurology, Ann & Robert H. Lurie Children's Hospital of Chicago, Chicago, IL; 2 Departments of Pediatrics and Neurology, Northwestern University Feinberg School of Medicine, Chicago, IL

**Keywords:** Neurology, Pediatrics, Child Development, Nervous System Diseases, Brain Diseases

## Abstract

Pediatric Neurology Briefs (PNB) has been published monthly since 1987 as a continuing education service designed to expedite and facilitate review of current medical literature concerning pediatric neurology. In 2015, PNB is relaunched as an open access, peer-reviewed, journal with an expanded editorial board. PNB has a new website and content management system capable of organizing peer-review and providing improved indexing, DOI assignment, and online full-text article view. Digitization of back issues, archiving, and inclusion in PubMed are future goals. The new online open access PNB aims to reach more physicians, researchers, and other healthcare providers with highlights of the latest advances in pediatric neurology and commentaries by specialists in the field.

*Pediatric Neurology Briefs* (PNB) has been published monthly since 1987 as a continuing education service designed to expedite and facilitate review of current medical literature concerning pediatric neurology. In 2015, PNB is relaunched as an open access, peer-reviewed, journal with an expanded editorial board. PNB has a new website and content management system capable of organizing peer-review and providing improved indexing, DOI assignment, and online full-text article view. Digitization of back issues, archiving, and inclusion in PubMed are future goals. The new online open access PNB aims to reach more physicians, researchers, and other healthcare providers with highlights of the latest advances in pediatric neurology and commentaries by specialists in the field.

PNB has reviewed and referenced articles from over 200 journals since inception. So far, PNB consists of 28 volumes with greater than 3200 articles, and includes 10,000 citations referencing the works of approximately 28,000 scholarly authors. Prior to the advent of the internet, the Editor periodically compiled and published the PNB articles in book form with index, according to subject heading and in chronological order [1, 2, 3].

In 2012, PNB became available online with the digital edition that was designed and introduced by Gordon T. Millichap, the interim Managing Editor. The new website allowed PNB to become a publishing partner with both the World Health Organization's HINARI Program and EIFL. These services provide equitable online access to the major journals in biomedical sciences for eligible not-for-profit health institutions and libraries, making PNB available in more than 100 developing countries, benefiting many thousands of health workers and researchers, and in turn, contributing to improve world health.

In 2015, the new editorial board will select articles, invite authors, and oversee peer-review of all submitted articles. Source article criteria include recent publication in a peer-reviewed journal and a topic of clinical value to practicing pediatric neurologists. PNB covers a comprehensive group of subject areas related to progress in pediatric neurology including, but not limited to, Seizure [4], Muscle [5], Metabolic [6], Headache [7], and Heredo-Degenerative Disorders [8]. Contributing Editors provide detailed summaries of published articles, followed by commentaries based on their experience and corroborated by appropriate supplementary citations. John J. Millichap becomes the journal Editor and is supported by J. Gordon Millichap, as Founding Editor, the Editorial Advisory Board, and invited expert Contributing Editors.

Despite the changes in editorial structure and distribution, the mission of PNB remains the same: “*Pediatric Neurology Briefs* is a continuing education service designed to expedite and facilitate the review of current scientific research and advances in child neurology and related subjects.” The Editors of PNB are indebted to the enduring support of subscribers to the print version for the past 28 years and look forward to the addition of new readers of the open access journal. Comments and suggestions from readers are welcome and may be sent to Editor at j-millichap@northwestern.edu.
